# Cannabinoid CB_2_ receptors in the mouse brain: relevance for Alzheimer’s disease

**DOI:** 10.1186/s12974-018-1174-9

**Published:** 2018-05-24

**Authors:** Alicia López, Noelia Aparicio, M. Ruth Pazos, M. Teresa Grande, M. Asunción Barreda-Manso, Irene Benito-Cuesta, Carmen Vázquez, Mario Amores, Gonzalo Ruiz-Pérez, Elena García-García, Margaret Beatka, Rosa M. Tolón, Bonnie N. Dittel, Cecilia J. Hillard, Julián Romero

**Affiliations:** 1 Laboratorio de Apoyo a la Investigación, Hospital Universitario Fundación Alcorcón, C/ Budapest 1, 28922 Alcorcón, Madrid Spain; 2 Universidad Rey Juan Carlos, Móstoles, Spain; 3 Faculty of Experimental Sciences, Universidad Francisco de Vitoria, 28223 Pozuelo de Alarcón, Madrid Spain; 4 Blood Research Institute, BloodCenter of Wisconsin, Milwaukee, WI 53226 USA; 5 Department of Pharmacology and Neuroscience Research Center, Medical College of Wisconsin, Milwaukee, WI 53226 USA

**Keywords:** Cannabinoid CB_2_ receptor, Transgenic mice, Enhanced green fluorescent protein, Amyloid, Neuroinflammation, Microglia

## Abstract

**Background:**

Because of their low levels of expression and the inadequacy of current research tools, CB_2_ cannabinoid receptors (CB_2_R) have been difficult to study, particularly in the brain. This receptor is especially relevant in the context of neuroinflammation, so novel tools are needed to unveil its pathophysiological role(s).

**Methods:**

We have generated a transgenic mouse model in which the expression of enhanced green fluorescent protein (EGFP) is under the control of the *cnr2* gene promoter through the insertion of an Internal Ribosomal Entry Site followed by the EGFP coding region immediately 3′ of the *cnr2* gene and crossed these mice with mice expressing five familial Alzheimer’s disease (AD) mutations (5xFAD).

**Results:**

Expression of EGFP in control mice was below the level of detection in all regions of the central nervous system (CNS) that we examined. CB_2_R-dependent-EGFP expression was detected in the CNS of 3-month-old AD mice in areas of intense inflammation and amyloid deposition; expression was coincident with the appearance of plaques in the cortex, hippocampus, brain stem, and thalamus. The expression of EGFP increased as a function of plaque formation and subsequent microgliosis and was restricted to microglial cells located in close proximity to neuritic plaques. AD mice with CB_2_R deletion exhibited decreased neuritic plaques with no changes in IL1β expression.

**Conclusions:**

Using a novel reporter mouse line, we found no evidence for CB_2_R expression in the healthy CNS but clear up-regulation in the context of amyloid-triggered neuroinflammation. Data from CB_2_R null mice indicate that they play a complex role in the response to plaque formation.

**Electronic supplementary material:**

The online version of this article (10.1186/s12974-018-1174-9) contains supplementary material, which is available to authorized users.

## Background

It has been long appreciated that cannabinoids such as ∆^9^-tetrahydrocannabinol (THC) exert effects on the immune system [40]. A primary target for the cannabinoids to alter immune system function, the cannabinoid receptor, subtype 2 (CB_2_R), was identified molecularly in 1993 [23]. Autoradiographic and in situ hybridization studies indicated a high level of expression of the CB_2_R in cellular elements of the immune system but these methods did not detect CB_2_R expression in the central nervous system (CNS) [12, 18]. According to these early data, the abundance of CB_2_R message in human blood cells was highest in B-lymphocytes, followed by natural killer cells, macrophages, and cluster of differentiation (CD)8 and CD4 T-lymphocytes [12].

The presence of CB_2_Rs in the CNS has been the subject of intense debate during the last decade. Some reports [13, 35] showed the expression of CB_2_Rs in neuronal elements of the uninjured brain, based primarily on immunohistochemical approaches. Other studies, however, limited the presence of CB_2_R in the CNS to glial cells and, specifically, to microglia [6]. Seminal studies by Cabral and colleagues suggested that CB_2_R could be expressed by microglial cells and that the expression level varied as a function of cell activation [9]. Subsequent studies confirmed this hypothesis [19, 31]. Regarding human samples, we found expression of CB_2_R was restricted to perivascular microglia in control brains [24] but that CB_2_R protein were dramatically increased in different pathological conditions. Observations made in Alzheimer’s disease (AD), multiple sclerosis, Down’s syndrome, and immunodeficiency virus-induced encephalitis confirmed that the presence of CB_2_R is greatly enhanced in areas of neuroinflammation, predominantly in microglial cells (see [6], for a review).

However, concerns regarding the lack of specificity of antibodies against the CB_2_R protein have been raised [3] (Additional file 1), which call into question some of these results. It is clear that additional tools are needed to unambiguously demonstrate the cellular expression of CB_2_R throughout the body, but most particularly within the CNS. We here introduce a novel transgenic model designed to unveil the functional distribution of cannabinoid CB_2_R and present data regarding the expression of these receptors in the mouse, with special attention to the CNS. Furthermore, we used this new mouse model to analyze the changes in the brain expression pattern of this receptor in the context of AD.

## Methods

### Generation of CB_2_^EGFP/f/f^ and CB_2_^−/−^ mice

**Fig. 1:**
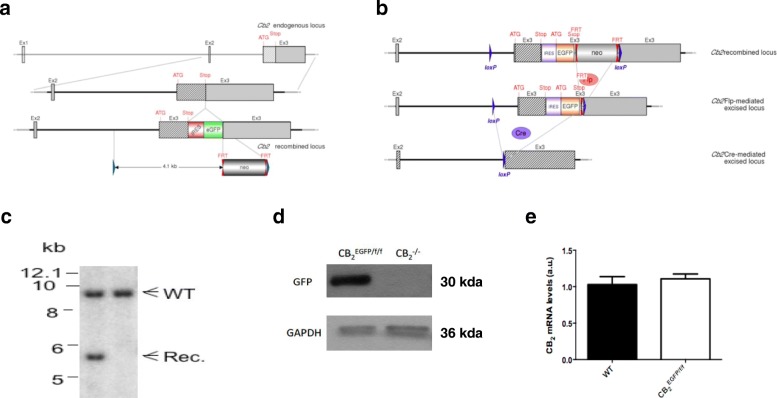
Generation of a novel mouse model. **a** Genomic structure of the construct employed for the generation of CB_2_^EGFP/f/f^ and CB_2_^−/−^ mice. Hatched rectangles represent *cnr2* coding sequences, gray rectangles indicate non-coding exon portions, solid lines represent chromosome sequences. The neomycin-positive selection cassette (Neo) IRES sequence and reporter gene (EGFP) are indicated. *lox*P sites are represented by blue triangles and FRT sites by double red triangles. The initiation (ATG) and Stop (Stop) codons are indicated. For the generation of knock-in mice (CB_2_^EGFP/f/f^), recombined mice were bred with ubiquitous FLP-recombinase expressing mice, enabling the deletion of the FRT-flanked region. **b** For the generation of knock-out mice (CB_2_^−/−^), recombined mice were bred with ubiquitous Cre-recombinase expressing mice, resulting in the deletion of the *lox*P-flanked region. **c** Representative Southern blot showing the expected wild-type (WT) and recombined (Rec) hybridization signals at 9302 and 5794 bp, respectively, in embryonic stem cells from non-transfected (right column) and successfully transfected clones (left column). **d** Representative Western blot showing EGFP expression in spleen tissue homogenates from CB_2_^EGFP/f/f^ (left lane) and CB_2_^−/−^ mice (right lane). **e** The basal expression level of CB_2_ receptor mRNA is not modified as a consequence of the insertion of the genetic construct used for the generation of the knock-in mice (CB_2_^EGFP/f/f^), as revealed by qRT-PCR in spleen samples and comparison with WT mice

Mice were generated at Genoway facilities (Lyon, France). A targeting strategy was designed consisting in the insertion of an enhanced Green Fluorescent Protein (EGFP) reporter gene, preceded by an Internal Ribosomal Entry Site (IRES) sequence in the 3′ untranslated region (UTR) of the *cnr2* mouse gene. This approach results in the expression of the reporter gene under the control of the endogenous mouse *cnr2* promoter and transcript from the same bicistronic mRNA as the CB_2_R protein. Further, the entire exon 3, including the 3′ UTR and knocked-in reporter, is flanked by *lox*P sites, allowing the conditional inactivation of the *cnr2* gene in cells expressing Cre recombinase (Fig. 1a).

Three isolated sequences encompassing the murine *cnr2* gene regions surrounding the targeted exon 3 were used for the construction of the targeting vector. These sequences included (i) a 3462 bp-sized fragment containing exon 2 and downstream intronic sequences, (ii) a 2980 bp-sized fragment containing the coding part of exon 3 and upstream intronic sequences, and (iii) a 3657 bp-sized fragment containing the non-coding part of exon 3 and downstream sequences. The linearized targeting construct was transfected into C57BL/6J embryonic stem cells. Homologous recombinant cells were identified by Southern analysis and five clones were used to generate chimeric mice. Chimeras were bred with C57BL/6J Flp- and Cre-deleter females, in order to generate Neo-excised EGFP reporter knock-in (CB_2_^EGFP/f/f^) mice (Fig. 1a) and constitutive knock-out (CB_2_^−/−^) mice (Fig. 1b), respectively.

Homozygous mice identified by PCR were further verified by Southern blot analysis (Fig. 1c). All mice used in this study were fourth- or fifth-generation offspring from intercrosses of C57BL/6J mice. Mice were housed and bred in the animal facilities of Universidad Rey Juan Carlos (Alcorcón, Madrid, Spain) or the Medical College of Wisconsin (Milwaukee, WI, USA). Experimental protocols met the European and Spanish regulations for protection of experimental animals (86/609/EEC and RD 1201/2005 and 53/2013) or were approved by the Institutional Animal Care and Use Committee of the Medical College of Wisconsin. Male mice were used in all experiments included in the present report with the exception of flow cytometry experiments (see below).

### Generation of CB_2_^EGFP/f/f^/5xFAD and CB_2_^−/−^/5xFAD mice

Mice co-expressing five familial Alzheimer’s disease mutations (5xFAD) were purchased from Jackson Laboratories (Bar Harbor, ME, USA; [25]) on the C57BL/6J background and were mated with CB_2_^EGFP/f/f^ and CB_2_^−/−^ mice and backcrossed for at least five generations to generate CB_2_^EGFP/f/f^/5xFAD and CB_2_^−/−^/5xFAD mice. Animals employed in the present experiments were 3 to 6 months old; this period was chosen based on previously published data [25, 36] in order to allow for the appearance of amyloid deposits.

### Flow cytometry

Single cell suspensions were prepared from the spleens of wild type, CB_2_^EGFP/f/f^, and CB_2_^EGFP/f/+^ mice of both sexes as described previously [27]. Cells were incubated with combinations of anti-mouse fluorescently-conjugated antibodies as follows: anti-B220 PE, anti-CD4 APC-eFluor780, anti-CD8 eFluor450, anti-CD11b eFluor450, anti-CD11c PE, anti-Ly6C APC, anti-Ly6G APC/Cy7, and anti-NK1.1 APC. Flow cytometry was used to identify B cells (B220^+^CD4^−^CD8^−^), CD4 T cells (CD4^+^NK1.1^−^), CD8 T cells (CD8^+^ NK1.1^−^), NKT cells (CD4^+^NK1.1^+^), NK cells (CD4^−^NK1.1^+^), macrophages (CD11b^+^Ly6C^+/−^Ly6G^−^), dendritic cells (CD11b^+^CD11c^hi^), and granulocytes (CD11b^+^Ly6C^+^Ly6G^+^). Sample acquisition was performed on a BD Biosciences LSR II, and data was analyzed using FlowJo software to generate the geometric mean of eGFP expression in each immune cell population.

### Immunofluorescence and neuritic plaque staining

Mice (*N* = 4–6 mice per group) were deeply anesthetized and transcardially perfused with cold PBS (pH 7.4) followed by freshly prepared cold 4% paraformaldehyde in PBS (pH 7.4). Tissue samples were collected and post-fixed in the same fixative overnight. Afterwards, tissues were dehydrated by sequential transfer to 10 and 30% sucrose solutions. Finally, tissues were cryoprotected with Tissue-Tek and frozen in dry ice. Thirty-micrometer-thick sections were obtained in a cryostat and preserved in cryoprotectant solution until use.

Floating tissue sections were washed with Tris Buffer Saline (TBS) before overnight incubation at 4 °C with the primary antibodies used for identification of the cellular types. For EGFP identification, overnight incubation with an anti-GFP antibody (1:1500; Abcam) was followed by incubation with an Alexa 488 anti-chicken antibody conjugate (Invitrogen) carried out at 37 °C for 2 h, rendering green fluorescence. Afterwards, sections were incubated with a rabbit polyclonal anti-ionized calcium-binding adaptor molecule 1 (Iba1) (1:1000 dilution, Wako, Osaka, Japan), diluted in TBS containing 1% bovine serum albumin (BSA; Sigma, St. Louis, USA) and 1% Triton x-100 (Sigma). After the incubation, sections were washed in TBS followed by incubation with an Alexa 546 anti-rabbit antibody conjugate (Invitrogen, Eugene, OR, USA) at 37 °C for 2 h, rendering red fluorescence. Additional tissue sections were incubated with mouse monoclonal anti-GFAP-Cy3 antibody (1:1500 dilution, Sigma) in the same buffer for 2 h at 37 °C or with mouse monoclonal anti-neuron-specific nuclear protein (NeuN) antibody (1:1000 dilution, Merck Millipore, Darmstadt, Germany) followed by incubation with Alexa 594 anti-mouse antibody conjugate (Invitrogen) as described above.

In order to study amyloid plaque deposits, a subset of CB_2_^EGFP/f/f^/5xFAD mice received an i.p. dose of 10 mg/kg of methoxy-XO4 (a Congo Red derivative known to selectively stain amyloid plaques; Tocris Bioscience; [4]) 24 h prior to sacrifice. Brains were processed and sections were obtained and preserved for immunostaining as described above.

Sections were mounted in aqueous solution (Vectashield, Vector Laboratories, Burlingame, CA, USA), coverslipped, and sealed. Slides were studied and photographed with upright microscopes (Nikon 90i, Nikon, Tokyo, Japan; and Axioimager M2, Zeiss, Oberkochen, Germany) and using a DXM1200F camera and C1 and LSM710 confocal systems [36]. Image analysis was carried out as described [36] with Metamorph (Molecular Devices, Sunnyvale, CA, USA) and ImageJ software (Research Services Branch, National Institute of Mental Health, Bethesda, MD, USA).

### Western blotting

Protein fractions were collected from a Tri-pure extraction of hippocampal and spleen tissues, according to the manufacturer’s instructions (Roche). Lysates (20 μg/lane or 10 μg/lane for hippocampal and spleen protein samples respectively) were separated by SDS-PAGE and transferred onto PVDF membranes (BioRad). After blocking in Tris-Tween buffered saline (TTBS; 10 mM Tris pH 7.5, 150 mM NaCl, 0.1% Tween 20 plus 5% nonfat dried milk), they were incubated overnight at 4 °C, as appropriate, with anti-GFP (1:500, Abcam, Cambridge, UK). Membranes were incubated with corresponding horseradish peroxidase-conjugated secondary antibody (1:8000) and were developed using a chemiluminescent reagent (ECL detection reagent GE Healthcare, Buckinghamshire, UK). Developed signals were recorded on X-ray film (Agfa) for densitometric analysis (ImageJ, NIH, MD, USA). *N* = 4–6 mice per group were used for protein quantification by Western blot.

### ELISA Aβ_1-42_

Human ELISA kits (Invitrogen, Camarillo, CA, USA) were used for the quantification of Aβ_1-42_ in the brain soluble fractions, following the instructions provided by the manufacturer. Levels were normalized to the total amount of protein.

### Real-time quantitative PCR for CB_2_ and IL1β

Total RNA was isolated using Tripure Isolation Reagent (Roche, Mannheim, Germany) according to the protocol of the supplier. RNA was dissolved in RNase-free water and quantified by absorption at 260 nm. Aliquots were subjected to 1% denaturing agarose gel electrophoresis and GelRed Nucleic Acid Gel Stain (Biotium, Fremont, CA, USA) staining to verify the quantity and quality of RNA. Single-stranded complementary DNA (cDNA) was synthesized from 1 mg of total RNA using LightCycler Taqman Master (Roche Diagnostics). PCR primers and TaqMan probes were designed by Tib Molbiol (Berlin, Germany) (see Additional file 2: Figure S1). For normalization, 18S primers and probe number 55 from Universal ProbeLibrary (Roche) were utilized. Gene expression was quantified using LightCycler FastStart DNA Master HybProbe and LightCycler Taqman Master (Roche) and Quantimix Easy Probes kit (Biotools, Madrid, Spain) in a LightCycler thermocycler (Roche). The concentration of primers and probes were 0.5 and 0.2 μM, respectively. PCR assays were performed using 2 μl of the cDNA reaction. All assays were carried out twice as independent PCR runs for each cDNA sample. Mean values were used for further calculation. A negative (no template) control was measured in each of the PCR runs. Standard curves were calculated for quantification purposes using fivefold serial dilutions of cDNA from mouse brain. The transcript amounts were calculated using the second derivate maximum mode of the LC-software version 4.0. The specific transcript quantities were normalized to the transcript amounts of the reference gene 18S. All further calculations and statistical analyses were carried out with these values referred to as relative expression ratios.

### Statistics

Results are expressed as mean ± SEM. Statistical analysis were made using student’s *t* test for comparisons between two groups, analysis of variance (ANOVA), and two-way ANOVA with Tukey’s post-test for multiple comparisons. A *p* value < 0.05 was considered as statistically significant (see Additional file 3: Table S1). Data were analyzed with Graph Pad Prism software version 6.0 (San Diego, CA, USA).

## Results

### Basal expression of EGFP in CB_2_^EGFP/f/f^ mouse spleen is coincident with previously described CB_2_ receptor patterns of expression in immune cells

To characterize the newly generated CB_2_^EGFP/f/f^ mice, we performed Western blotting on spleen samples. A single band corresponding to the EGFP molecular weight was evident in CB_2_^EGFP/f/f^ mice and was undetectable in spleen samples from CB_2_^−/−^ mice (Fig. 1d). We determined whether the strategy for the generation of the knock-in mice modified the expression levels of CB_2_R gene. Our results show that no changes were evident in CB_2_R mRNA expression levels between WT and CB_2_^EGFP/f/f^ mice in spleen (Fig. 1e; *p* = 0.474), thus ruling out a putative impact of the transgene on basal expression of the receptor.

**Fig. 2:**
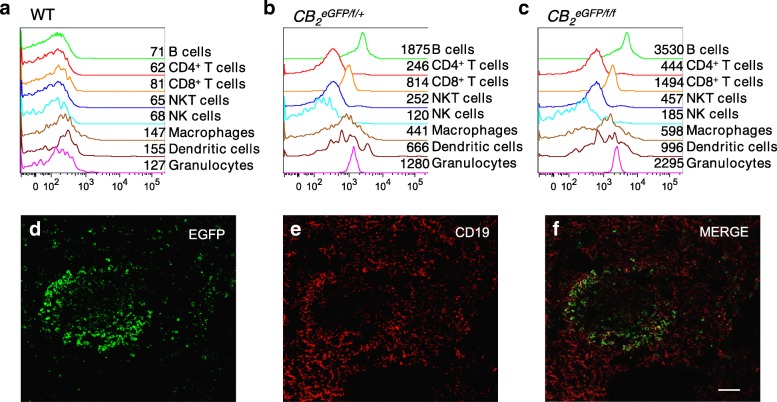
EGFP presence and distribution in CB_2_^EGFP/f/^ spleens determined using flow cytometry (**a**–**c**) and immunofluorescence (**d**–**f**). **a**–**c** Determination of cell-specific expression of EGFP in splenocytes harvested from WT (**a**), CB_2_^EGFP/f/+^ (**b**), and CB_2_^EGFP/f/f^ (**c**) mice. The numbers next to the histograms are the mean fluorescence intensity (MFI). **d**–**f** CB_2_-dependent-EGFP expression in B-lymphocytes in the spleen of CB_2_^EGFP/f/f^. EGFP expression was evident in follicles of the white pulp. Scale bars, 100μm (**d**–**g**)

We used flow cytometry to identify and quantify the EGFP expression of splenocyte cell populations from wild type, CB_2_^EGFP/f/+^, and CB_2_^EGFP/f/f^ mice (Fig. 2a–c). Using wild type mice, we found that background EGFP immunofluorescence was low in all immune cell populations examined (Fig. 2a). EGFP expression levels in splenic immune cells were compared in heterozygous (Fig. 2b) and homozygous (Fig. 2c) CB_2_^EGFP/f/f^ mice. In all immune cell populations investigated, the homozygous CB_2_^EGFP/f/f^ mice exhibited approximately double the mean fluorescence intensity (MFI) of the heterozygous mice. EGFP expression was highest in the B cell population, which is consistent with reports that B cells have the highest CB_2_ receptor expression among these cell types [12]. Among the T cell populations, CD4 T cells and NK T cells expressed a similar low level of EGFP expression, while CD8 T cells expressed ~ threefold higher levels (Fig. 2). NK cells expressed negligible levels of EGFP (Fig. 2). Monocytes/macrophages and dendritic cells expressed EGFP in a broader expression pattern than the lymphocytes (Fig. 2). Given that the spleen contains numerous macrophage and dendritic cell populations, it is likely that CB_2_R, and thus EGFP, will be differentially expressed among them [7, 15]. Finally, of the myeloid subset, granulocytes exhibited the highest amount of EGFP expression. These data are consistent with the published reports of CB_2_R distribution among these cell types [12] indicating that the CB_2_^EGFP/f/f^ mouse is an excellent tool by which to determine steady state CB_2_R expression in various spleen cell populations using EGFP fluorescence.

We analyzed the expression of EGFP in spleens of CB_2_^EGFP/f/f^ mice by immunofluorescence and found discrete cell populations showing detectable signal. EGFP^+^ B cells were detected, limited to the marginal zone of the white pulp follicles, mostly located in the follicular corona (Fig. 2d–f).

### Basal expression of EGFP in CB_2_^EGFP/f/f^ mice is undetectable in the CNS but is induced as a consequence of amyloid deposition

**Fig. 3:**
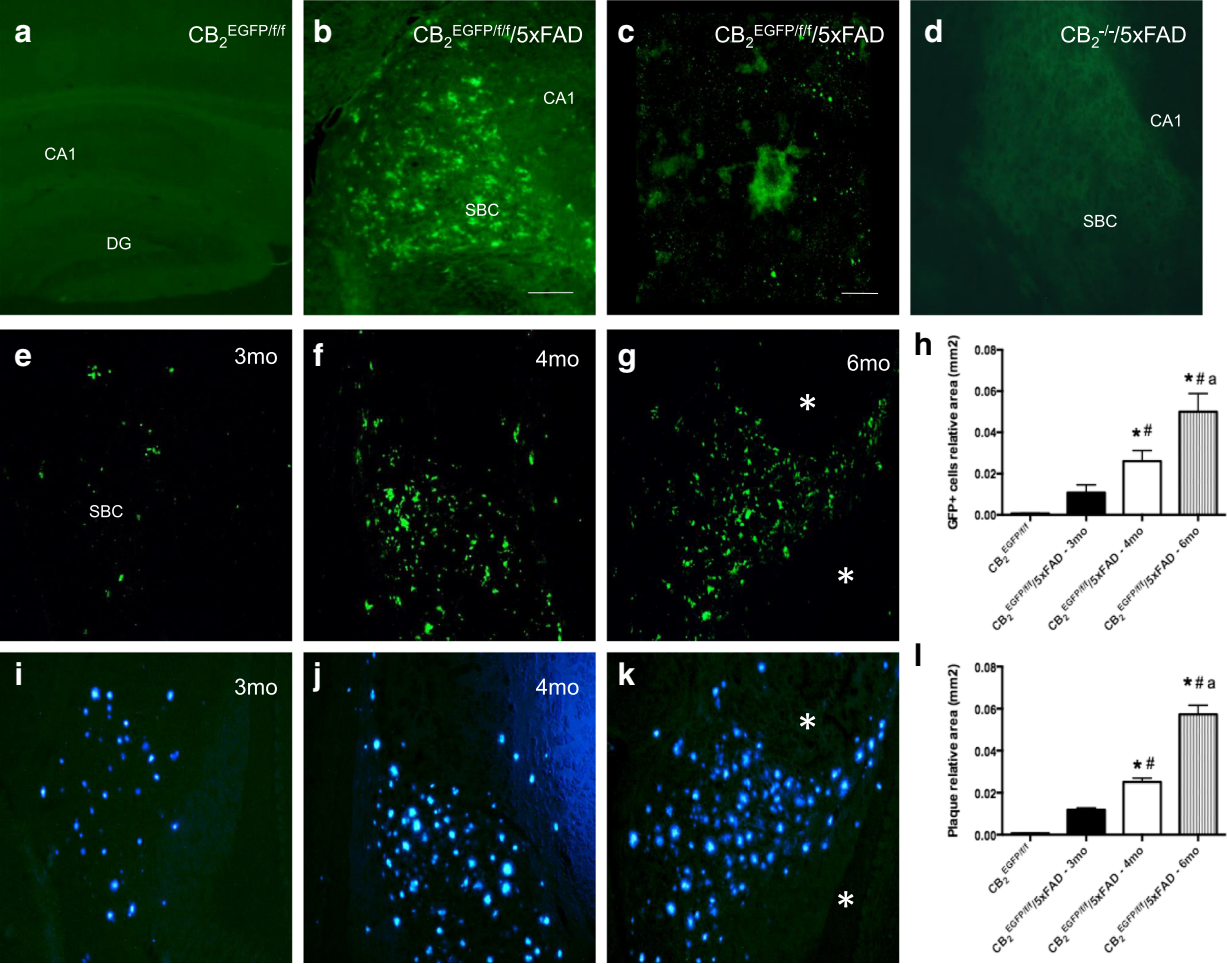
In vivo EGFP induction in the context of AD, as revealed by immunofluorescence. **a** Un-manipulated, healthy CB_2_^EGFP/f/f^ mice showed no significant EGFP signal in hippocampus (**a**). **b**, **c** EGFP signal could be noticed in the brain of CB_2_^EGFP/f/f^/5xFAD mice; these cells showed ameboid shape. **d** No EGFP signal could be observed in any brain region of CB_2_^−/−^/5xFAD mice, including those enriched in amyloid plaques such as hippocampus. **e**–**l** EGFP signal in CB_2_^EGFP/f/f^/5xFAD mice increased with age and paralleled that of amyloid deposits. EGFP was evident in brain samples of CB_2_^EGFP/f/f^/5xFAD mice starting at 3 months of age (**e**) and progressively increasing with age (4 months, **f**, and 6 months, **g**) and matched with the pattern of distribution of amyloid-enriched plaques stained with methoxy-XO4 in those same samples (figure **i**–**k**). Note that neighboring brain regions devoid of amyloid deposits exhibited a complete absence of EGFP signal (asterisks in **g**, **k**). **h**, **l** Densitometric quantification of EGFP^+^ cells (**h**) and amyloid plaques (**k**) shows a parallel increase in neuritic plaques and EGFP expression. Data are expressed as mean ± SEM. *p < 0.05 vs CB_2_^EGFP/f/f^ mice, # vs CB_2_^EGFP/f/f^/5xFAD-3mo mice, and “a” vs CB_2_^EGFP/f/f^/5xFAD-4mo mice. *N* = 5 for immunofluorescence labeling. Scale bars, 10 μm (**c**) and 50 μm. DG (dentate gyrus); SBC (subiculum); CA1 (CA1 region of Ammon’s horn)

In the CNS, microscopic analysis of the brain and spinal cord of 3-, 4-, or 6-month-old CB_2_^EGFP/f/f^ mice showed no detectable EGFP immunoreactivity above background in glial or neuronal elements of any region examined, which included hippocampus (Fig. 3a), cortex, cerebellum, thalamus, brain stem, and spinal cord (not shown). In contrast, intense EGFP signal could be seen in brain regions of CB_2_^EGFP/f/f^/5xFAD mice known to be rich in beta-amyloid neuritic plaques, such as hippocampus (Fig. 3b). Other regions such as cortex, thalamus, and brain stem also exhibited EGFP signal (data not shown), in concordance with the previously reported distribution of neuritic plaques [25]. EGFP^+^ cells exhibited an ameboid shape and were mostly found in clusters (Fig. 3c), suggesting they could be activated microglial cells. No signal could be observed in the hippocampus of CB_2_^−/−^/5xFAD mice (Fig. 3d) or in any other brain region examined (data not shown).

As shown in Fig. 3e, EGFP immunoreactivity above background could be observed as early as 3 months of age in the CB_2_^EGFP/f/f^/5xFAD mice, and EGFP-labeled cells increased in density with age in these mice (Fig. 3e–h). EGFP^+^ were found in clusters throughout the brain parenchyma and their distribution and increased density with age paralleled that of neuritic plaques, identified using methoxy-XO4, a dye for amyloid deposits (Fig. 3i–l). Interestingly, no EGFP signal could be observed in regions not exhibiting neuritic plaques (asterisks in Fig. 3g–k). The number of EGFP^+^ cells was dramatically increased at 4 and 6 months of age, which also paralleled the increase in the appearance of amyloid deposits (Fig. 3h: *F*_3,18_ = 58.46, *p* < 0.0001; Fig. 3l: *F*_3,23_ = 64.70, *p* < 0.0001).

### CB_2_R induction is limited to plaque-associated microglial cells

**Fig. 4:**
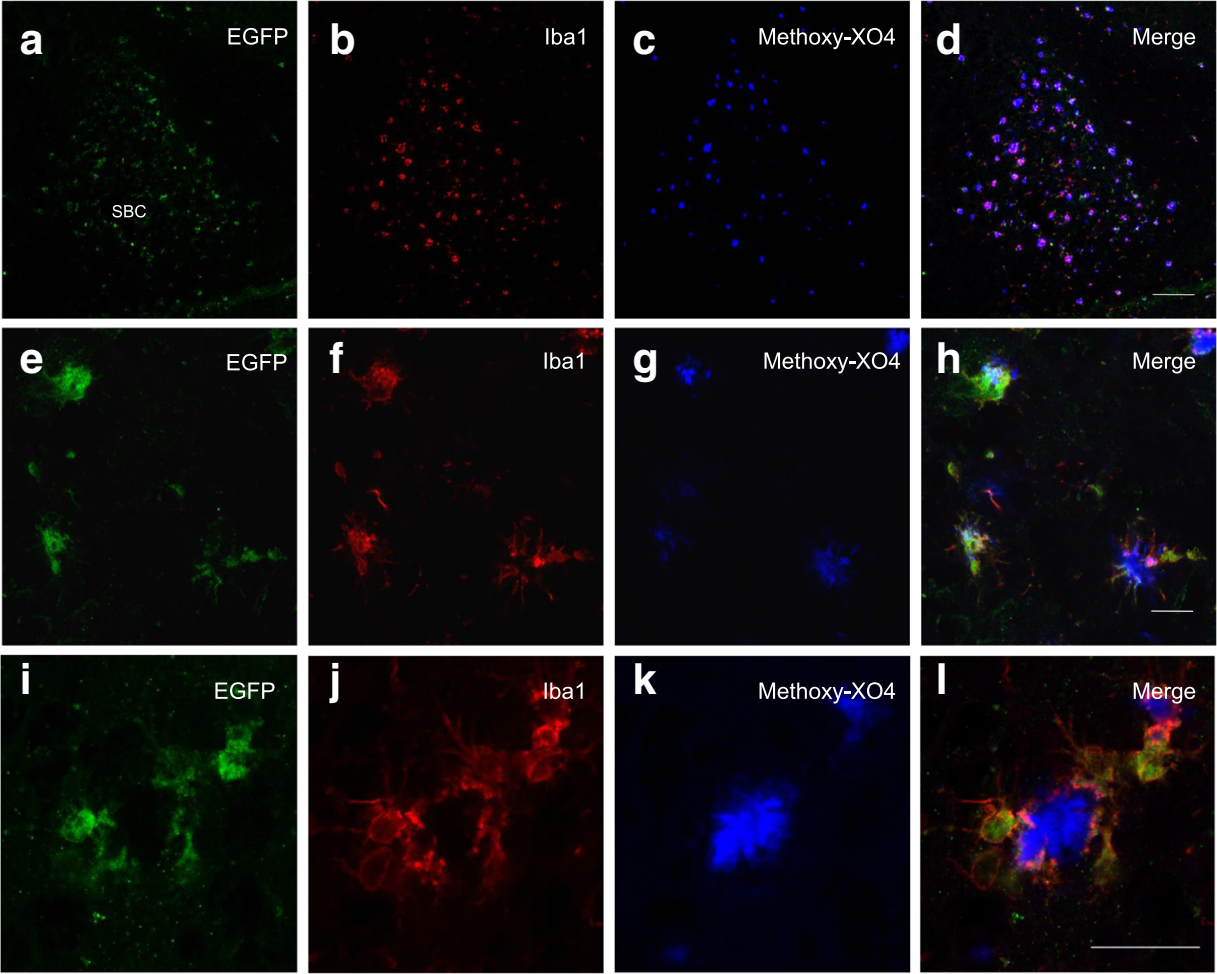
Restricted EGFP expression in microglial cells located in peri-plaque areas of the CB_2_^EGFP/f/f^/5xFAD mouse hippocampus. **a**–**l** Low-magnification photographs of EGFP^+^ microglial cells (**a**, **b**) in close association to beta-amyloid neuritic plaques (**c**, **d**). Medium- (**e**–**h**) and high-magnification (**i**–**l**) photographs of EGFP^+^ microglial cells. Detailed co-localization immunofluorescent analysis reveals a complete overlap between EGFP^+^ cells and Iba1^+^ cells, indicative of their macrophage/microglia nature, and a selective association to amyloid-enriched plaques (stained with methoxy-XO4). Scale bars, 100 μm (**a**–**d**), 25 μm (**e**–**h**), and 25 μm (**i**–**l**). SBC (subiculum)

**Fig. 5:**
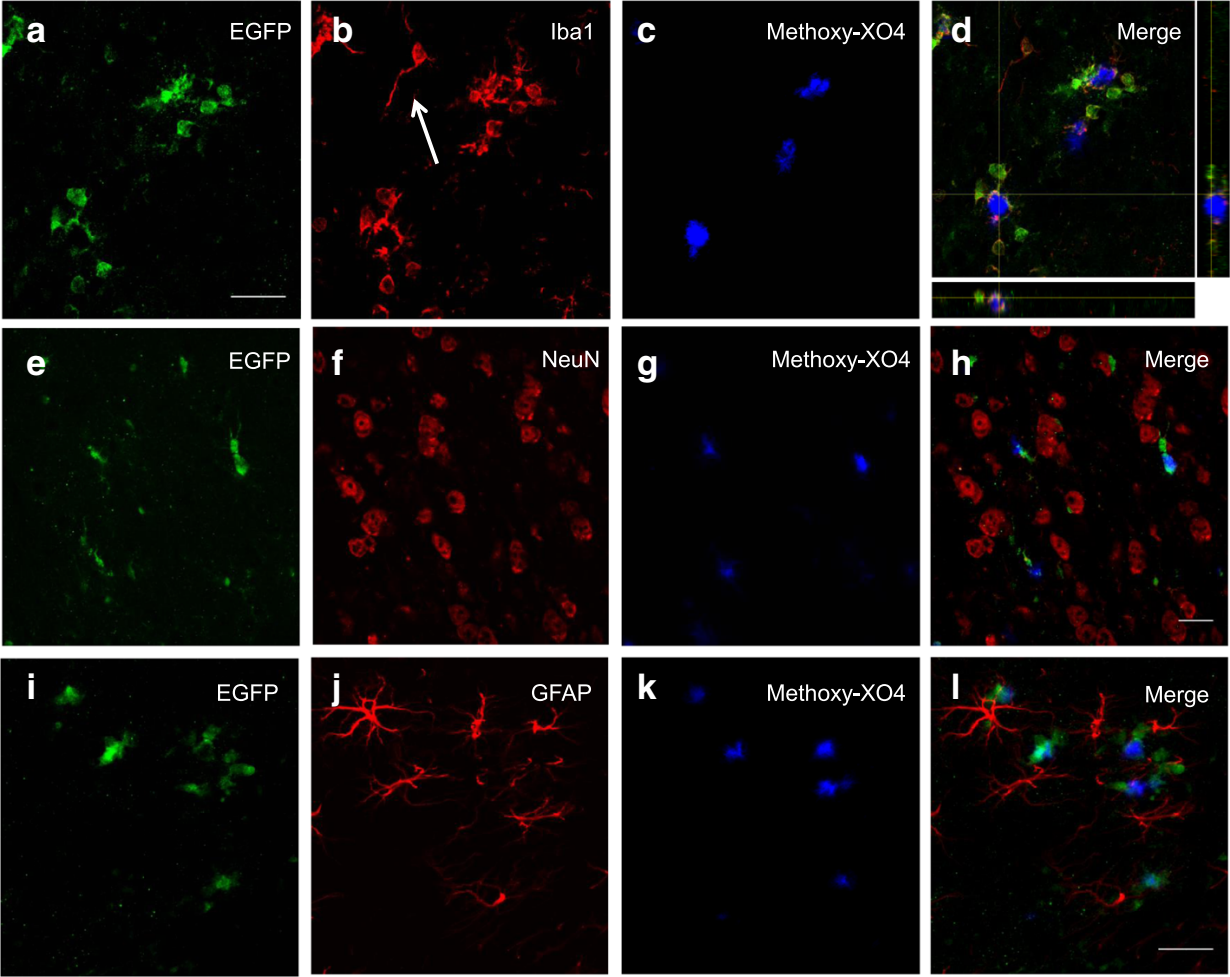
EGFP expression is limited to plaque-associated microglial cells but is absent in neurons and astrocytes in CB_2_^EGFP/f/f^/5xFAD mice. **a**–**c** Z-stack showing that EGFP expression (**a**) was evident in microglial cells (**b**) located in close association to amyloid-enriched neuritic plaques, as revealed by methoxy-XO4 (**c**). However, microglial cells not linked with these pathological structures (arrow in **b**) showed reduced EGFP signal. **d** Orthogonal view in *Z* axis of the cluster of microglial EGFP^+^ cells shown in (**a**–**c**). Note the intimate contact established by microglial processes into the neuritic plaque. **e**–**l** Neurons (NeuN^+^ cells; **e**–**h**) nor astrocytes (GFAP^+^ cells; **i**–**l**) showed no EGFP signal. Scale bars, 25 μm

EGFP^+^ cells were located in association with neuritic plaques (as revealed by staining with methoxy-XO4) and exhibited morphological features of microglia (Fig. 4). Co-localization studies with Iba-1, a commonly used marker of cells of myeloid lineage, were carried out. Low magnification (a to d) images showed a match in the pattern of distribution among EGFP^+^ and Iba1^+^ cells in the subiculum of 6-month-old CB_2_^EGFP/f/f^/5xFAD mice; in addition, our data show that CB_2_-dependent EGFP expression takes place selectively in Iba1^+^ cells located in the vicinity of neuritic plaques (Fig. 4e–l). Microglial cells not associated with these pathological structures showed no EGFP staining (see Fig. 5a–d). For example, note the microglial cell at the arrow in Fig. 5b is neither EGFP positive nor associated with a plaque. Differences in the morphological features of EGFP^+^ and EGFP^−^ microglial cells were evident, with EGFP^+^ cells exhibiting an ameboid-like shape (Fig. 5a and b), typical of activated microglia, while EGFP^−^ cells showed a highly ramified morphology, characteristic of quiescent, non-activated, microglia (arrow in Fig. 5b).

Furthermore, we also studied whether other cell types in the CNS, such as neurons or astrocytes expressed EGFP in CB_2_^EGFP/f/f^/5xFAD mice. To that end, co-localization studies with a neuronal marker (NeuN; Fig. 5e–h) or with a marker of astrocytes (GFAP; Fig. 5i–l) were carried out. Our data indicate that neither of these cell types express EGFP; thus, *cnr2*-dependent EGFP expression is limited to microglial cells in CB_2_^EGFP/f/f^/5xFAD mice.

### Changes associated with CB_2_R deletion include decreases in plaque deposition and no changes in gliosis or IL1β expression

**Fig. 6:**
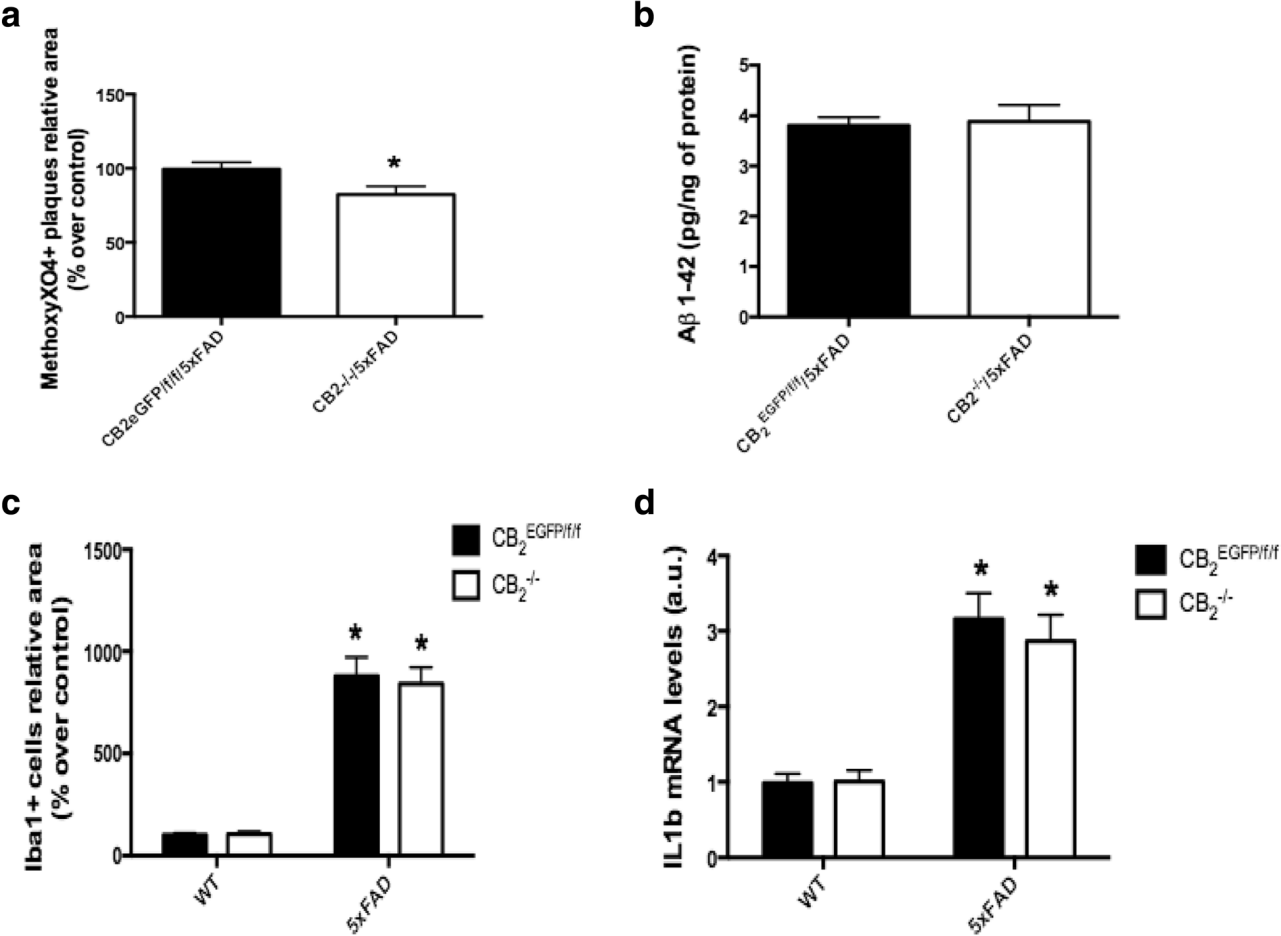
Consequences of CB_2_ deletion in the context of AD. The genetic inactivation of CB_2_ in CB_2_^−/−^/5xFAD mice led to a significant decrease in plaque density in hippocampus (**a**), without any changes in soluble amyloid production (**b**), or microgliosis (**c**). No changes were observed in the expression of IL1β as a consequence of gene deletion (**d**) in CB_2_^−/−^/5xFAD mice as compared to those in CB_2_^EGFP/f/f^/5xFAD mice. Data are expressed as mean ± SEM. *p < 0.05. Student’s *t*-test (**a**, **b**) and two-way ANOVA followed by Tukey’s post hoc test (**c**, **d**). *N* = 5 for immunofluorescence labeling measurements and *N* = 6 for experiments on soluble amyloid quantification and IL1β expression

We analyzed the impact of *cnr2* gene deletion on plaque formation, soluble amyloid levels and neuroinflammation (Fig. 6). We found a small but significant decrease in hippocampal neuritic plaque density (measured by staining with methoxy-XO4; Fig. 6a: *p* < 0.0338) in the CB_2_^−/−^ mice that was not paralleled by changes in soluble levels of Aβ_1-42_ in the hippocampus (measured by ELISA; Fig. 6b: *p* < 0.6413). Hippocampal microgliosis was assessed by counting Iba1^+^ cells in tissue sections. As expected, the 5xFAD mice exhibited a significant increase in Iba1^+^ cells (Fig. 6c: *F*_1,23_ = 85.84, *p* < 0.0001); however, there was no difference in this measure between the wild type and CB_2_^−/−^ mice (Fig. 6c: *F*_1,23_ = 0.03775, *p* = 0.8476). Finally, a significant increase in interleukin-1 beta (IL1β) was observed as a consequence of the amyloid pathology (Fig. 6d: *F*_1,23_ = 49.12, *p* < 0.0001) but CB_2_R genotype had no effect (*F*_1,33_ = 0.2229, *p* = 0.6400).

## Discussion

We have established a novel transgenic mouse model (CB_2_^EGFP/f/f^) that allows for identification of cells that are actively transcribing the *cnr2* gene. The use of an IRES allows for coupling of EGFP expression to *cnr2* gene transcription without loss or modification of the CB_2_ protein, which is a different approach from another reporter mouse line in which the *cnr2* gene is replaced by EGFP, resulting in a CB_2_R knock out [29, 30]. The present reporter mice are expected to provide crucial information on the distribution, expression, and pathophysiological roles of the CB_2_R, while maintaining its appropriate cellular expression. By crossing these mice with 5xFAD mice, we have expanded our knowledge regarding the relevance of CB_2_R in amyloid pathology. The main conclusions of this study are that, if CB_2_R are expressed by neurons or glia in the CNS of healthy, un-manipulated mice, they are expressed at very low turnover rates because no specific EGFP signaling could be detected in any region of the mouse brain or spinal cord. Second, under chronic neuroinflammatory stimuli (such as those derived from the deposition of the amyloid peptide in the brain parenchyma), the expression of CB_2_R is induced in microglial cells, and this induction takes place specifically in activated microglial cells surrounding neuritic plaques. These data confirm and expand previously published literature and support the contention that the presence of CB_2_R may be a diagnostic marker of neuroinflammation in the context of AD [5, 6] and other pathological conditions with a neuroinflammatory component [19, 20].

As previously suggested by us and by others [5, 19, 26], the expression of CB_2_R is induced under neuroinflammatory conditions in the human brain, being restricted to microglial cells closely associated to foci of neuroinflammation. Data obtained from samples of humans affected by several neurodegenerative conditions with accompanying neuroinflammation (i.e., AD, MS, HIV-encephalitis) revealed a consistent pattern of CB_2_R induction in microglia [6]. Our present data expand and confirm these observations. We used a well-known mouse model of amyloid pathology (5xFAD) to calibrate the impact that the appearance of neuritic plaques in the brain parenchyma has on the expression of CB_2_R. The analysis of CB_2_^EGFP/f/f^/5xFAD mice brain tissues showed that *cnr2*-dependent EGFP expression is present in microglial (Iba1^+^) cells located in the vicinity of amyloid-enriched neuritic plaques (as revealed with methoxy-XO4 in vivo staining). There was a remarkable lack of detectable EGFP expression in non-plaque areas. These data strongly support the hypothesis that CB_2_R gene expression is increased primarily in microglia that surround neuritic plaques.

The time-course of the appearance of neuritic plaques in the subiculum of CB_2_^EGFP/f/f^/5xFAD mice closely matched that previously described [25, 36]. Importantly, EGFP was detectable in plaque-associated microglia at 3 months of age, corresponding to the age when amyloid deposits are first present in the brain parenchyma. These data are indicative of the need to reach a threshold of inflammatory stimuli in the cellular milieu before the induction of CB_2_R expression takes place in the CNS. The present data suggest that threshold is reached coincident with appearance of the amyloid deposits. This suggests (i) that the induction of the expression of CB_2_ receptors takes place after a period of sustained inflammation and (ii) that CB_2_ receptors may be postulated as early markers of AD pathology. In this sense, it is important to note that disease-linked symptoms in 5xFAD mice are not evident before 6 months of age; thus, the induction of the *cnr2* gene expression is previous to phenotypic changes due to amyloid pathology, indicating that CB_2_R may provide diagnostic and therapeutic targets for the treatment of early stage AD [28].

CB_2_R functions in microglia as well as in other types of immune cells have been studied [8, 20]. In the context of AD neuroinflammation, there is evidence that CB_2_R agonists induce anti-inflammatory actions [1, 10, 21, 22, 26, 34], promote microglial migration and proliferation [37], and enhance amyloid removal [33, 38]. Furthermore, there is evidence that the activation of CB_2_R also decreases the production of amyloid peptides in a mouse model of AD [2], though conflicting results have been reported [29]. These effects make microglial CB_2_R interesting targets in amyloid-induced neuroinflammation as microglia play critical roles in the progression of the disease by modulating, for instance, amyloid removal, cytokine production or exosome-mediated peptide degradation [14].

Surprisingly in light of earlier studies, CB_2_^−/−^/5xFAD mice exhibited a small but significant decrease in neuritic plaque density in hippocampus compared to wild type 5xFAD mice that was not accompanied by a decrease in soluble Aβ_1-42_ levels, reduced microgliosis, or changes in IL1β expression. We do not have a conclusive explanation for this observation, though it is suggestive of a role for CB_2_R in microglial functions related to amyloid removal such as, for instance, phagocytosis [33]. In addition, conflicting results have been reported by several groups regarding the consequences of CB_2_ deletion on microgliosis, with both decreased and unchanged microgliosis being reported [2, 16, 30]. Further experiments are needed to clarify the reasons for these discrepancies regarding the impact of CB_2_R deletion on the formation of amyloid-enriched plaques.

Several recent studies indicate that CB_2_R agonists affect neuronal function [11, 32, 39]. In particular, CB_2_R agonists have been reported to affect hippocampal plasticity, effects that are lost in CB_2_^−/−^ mice. These results are difficult to reconcile in light of the lack of detectable EGFP in the hippocampus of the present transgenic mice and in another reporter model [29]. It is possible that the turnover of the CB_2_R in neurons is slower than the turnover of EGFP protein or the detectable amount of EGFP expression may be lower than the CB_2_R expression levels required to achieve a functional response in vivo. Alternatively, it is possible that CB_2_R expression is upregulated by the processes involved in the preparation of tissues for study ex vivo.

Our data are discordant compared to those reported in the Allen Mouse Brain Atlas [17]. Information provided by this platform reveals low but detectable levels of CB_2_-mRNA in olfactory and cortical subplate areas, as shown by single cell in situ hybridization (ISH). However, neither CB_2_^EGFP/f/f^ nor CB_2_^EGFP/f/f^/5xFAD mice showed specific EGFP signal in either of these regions. We do not have an explanation for this discrepancy other than the mentioned mismatch between detection limits, in this case referred to single cell-ISH (Allen Atlas) and EGFP immunostaining (present data).

## Conclusions

In summary, the present findings confirm and expand previous data showing the selective induction of CB_2_R in neuritic plaque-associated microglia and postulate these receptors as diagnostic and therapeutic targets in AD. The newly developed transgenic mouse model will be instrumental for elucidating their role(s) in neuroinflammatory conditions.

## Additional files

Additional file 1: CB2 Western blots. Test of different CB2 primary antibodies in spleen samples (with high CB2 expression levels in normal conditions) harvested from CB2EGFP mice (lines 1, 2, and 3) and CB2KO mice (lines 4, 5, and 6). GFP and beta-actin immunodetection was employed as internal controls. (PPTX 6471 kb)

Additional file 2: **Figure S1.** Sequences of the primers employed in the present studies. (DOCX 13 kb)

Additional file 3: **Table S1.** Statistical analysis of the data provided in the manuscript. (DOCX 17 kb)

## Abbreviations

5xFAD: Mice co-expressing five familial Alzheimer’s disease mutations
AD: Alzheimer’s disease
BSA: Bovine serum albumin
CB_2_R: Cannabinoid receptor, subtype 2
CD4: Cluster of differentiation 4
CNS: Central nervous system
EGFP: Enhanced green fluorescent protein
ELISA: Enzyme-linked immunosorbent assay
GFAP: Glial fibrillary acidic protein
i.p.: Intraperitoneal
Iba1: Ionized calcium-binding adaptor molecule 1
IL1β: Interleukin-1 beta
IRES: Internal Ribosomal Entry Site
ISH: In situ hybridization
NeuN: Neuron-specific nuclear protein
PBS: Phosphate buffered saline
PVDF: Polyvinylidene fluoride
qRT-PCR: Quantitative real-time polymerase chain reaction
SDS-PAGE: Sodium dodecyl sulfate polyacrylamide gel electrophoresis
THC: ∆^9^-tetrahydrocannabinol
TTBS: Tris-Tween buffer saline
UTR: Untranslated region
