# Supplementary material for: Cannabinoid CB2 receptors in the mouse brain: relevance for Alzheimer’s disease
Source: J Neuroinflammation. 2018 May 24;15:158. doi: 10.1186/s12974-018-1174-9 (PMC5968596; doi:10.1186/s12974-018-1174-9)
Supplement: Supplementary file 1 — CB2 Western blots. Test of different CB2 primary antibodies in spleen samples (with high CB2 expression levels in normal conditions) harvested from CB2EGFP mice (lines 1, 2, and 3) and CB2KO mice (lines 4, 5, and 6). GFP and beta-actin immunodetection was employed as internal controls. (PPTX 6471 kb) [file 12974_2018_1174_MOESM1_ESM.pptx]

## Slide 1
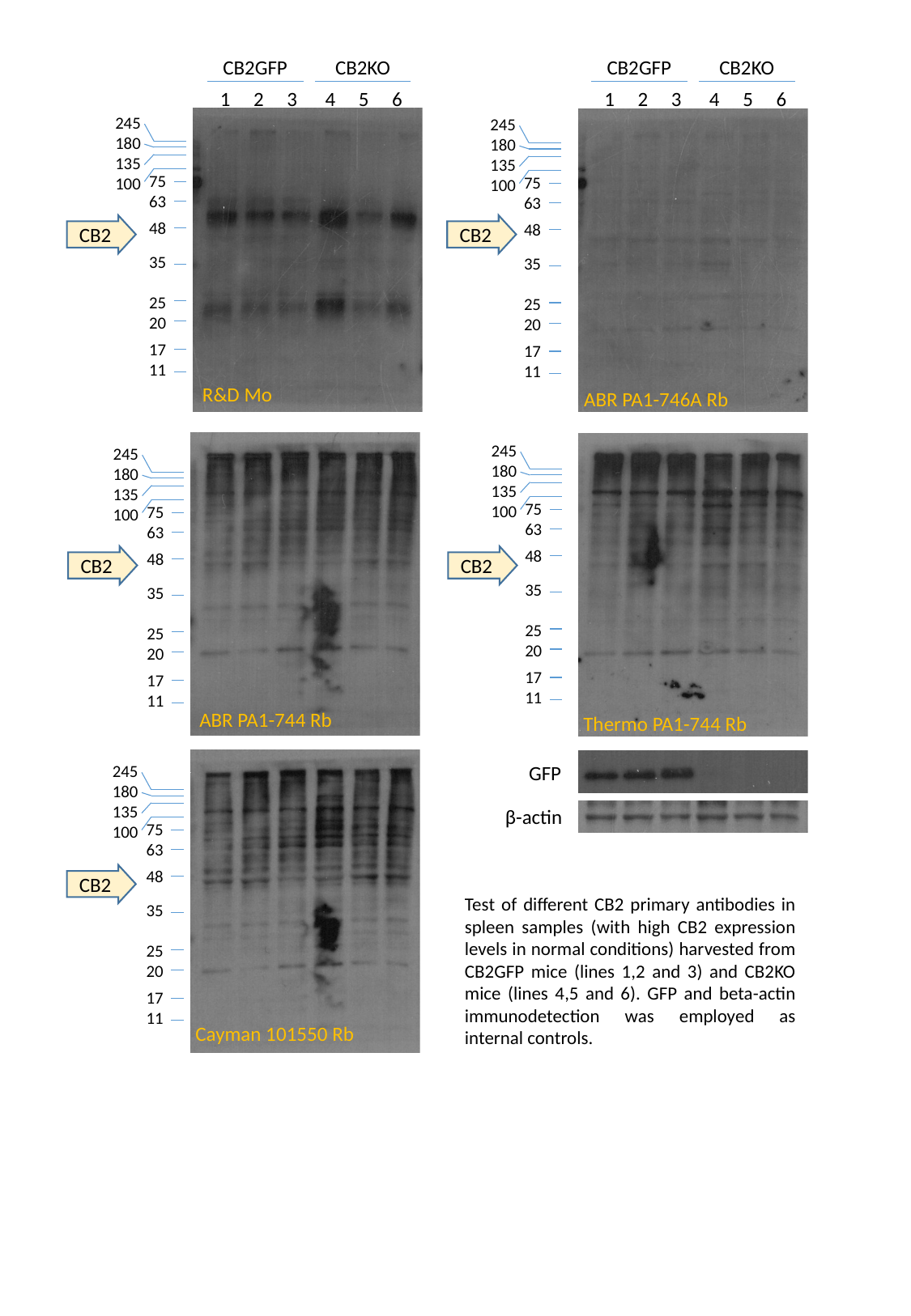

CB2GFP
CB2KO
CB2GFP
CB2KO
1 2 3 4 5 6
1 2 3 4 5 6
245
180
135
100
75
63
35
25
20
48
17
11
R&D Mo
245
180
135
100
75
63
35
25
20
48
17
11
ABR PA1-746A Rb
CB2
CB2
245
180
135
100
75
63
35
25
20
48
17
11
ABR PA1-744 Rb
245
180
135
100
75
63
35
25
20
48
17
11
Thermo PA1-744 Rb
CB2
CB2
245
180
135
100
75
63
35
25
20
48
17
11
Cayman 101550 Rb
GFP
β-actin
CB2
Test of different CB2 primary antibodies in spleen samples (with high CB2 expression levels in normal conditions) harvested from CB2GFP mice (lines 1,2 and 3) and CB2KO mice (lines 4,5 and 6). GFP and beta-actin immunodetection was employed as internal controls.
