# Supplementary material for: Cannabinoid CB2 receptors in the mouse brain: relevance for Alzheimer’s disease
Source: J Neuroinflammation. 2018 May 24;15:158. doi: 10.1186/s12974-018-1174-9 (PMC5968596; doi:10.1186/s12974-018-1174-9)
Supplement: Supplementary file 2 — Figure S1. Sequences of the primers employed in the present studies. (DOCX 13 kb) [file 12974_2018_1174_MOESM2_ESM.docx]

Additional file 2: Figure S1: Sequences of the primers employed in the present studies.

**CB2_A_**_:_ 5’- TCT CTC TTC GAG GGA GTG AAC TGA ACG-3’

**CB2_S_**: 5’- CTC GGT TAC AGA AAC AGA GGC TGA TG-3’

**IL1ß probe**: 5´-/56-FAM/AGTCACAGA/ZEN/GGATGGGCTCTTCTTCA/3IABkGQ/-3´

**IL1ß_S:_** 5´-TGT CTA ATG GGA ACG TCA CAC-3´

**IL1ß_A_**_:_ 5´- CAG GAT GAG GAC ATG AGC AC-3´
