# Supplementary material for: Cannabinoid CB2 receptors in the mouse brain: relevance for Alzheimer’s disease
Source: J Neuroinflammation. 2018 May 24;15:158. doi: 10.1186/s12974-018-1174-9 (PMC5968596; doi:10.1186/s12974-018-1174-9)
Supplement: Supplementary file 3 — Table S1. Statistical analysis of the data provided in the manuscript. (DOCX 17 kb) [file 12974_2018_1174_MOESM3_ESM.docx]

Additional file 3: Table S1: Statistical analysis of the data provided in the manuscript.

|  | **Factor** | **F(DFn, DFd), p value** |
| --- | --- | --- |
| *cnr2* gene (figure 1E) | Genotype | Mann-Whitney test, *p*=0.474 |
| EGFP^+^ cells (figure 3H) | Age | *F*_3,18_ = 58.46, *p*<0.0001 |
| Plaque relative area (figure 3L) | Age | *F*_3,23_ = 64.70, *p*<0.0001 |
| Plaque relative area (figure 6A) | Genotype | Mann-Whitney test, *p*<0.0338 |
| Aβ_1-42_ levels (figure 6B) | Genotype | Mann-Whitney test, *p*<0.6413 |
| Iba1^+^ cells (figure 6C) | WT vs 5xFAD | *F*_1,23_ = 85.84, *p*<0.0001 |
|  | CB_2_^EGFP/f/f^/ vs CB_2_^-/-^ | *F*_1,23_ = 0.03775, *p*=0.8476 |
|  | Interaction | *F*_1,23_ = 0.06376, *p*=0.8029 |
| *IL1β* gene (figure 6D) | WT vs 5xFAD | *F*_1,33_ = 49.12, *p*<0.0001 |
|  | CB_2_^EGFP/f/f^/ vs CB_2_^-/-^ | *F*_1,33_ = 0.2229, *p*=0.6400 |
|  | Interaction | *F*_1,33_ = 0.3000, *p*=0.5876 |
